# Associations of Serum Manganese Levels with Prediabetes and Diabetes among ≥60-Year-Old Chinese Adults: A Population-Based Cross-Sectional Analysis

**DOI:** 10.3390/nu8080497

**Published:** 2016-08-13

**Authors:** Xuan Wang, Mingyue Zhang, Guang Lui, Hong Chang, Meilin Zhang, Wei Liu, Ziwei Li, Yixin Liu, Guowei Huang

**Affiliations:** 1 Department of Nutrition and Food Science, School of Public Health, Tianjin Medical University, 22 Qixiangtai Road, Heping District, Tianjin 300070, China; wangxuan@tmu.edu.cn (X.W.); defjmmm@163.com (M.Z.); lzw30706sky@163.com (Z.L.); tjmulyx@126.com (Y.L.); 2 Department of Physics and Chemistry, Tianjin Centers for Disease Control and Prevention, 6 Huayue Road, Hedong District, Tianjin 300070, China; zhmyue@163.com (M.Z.); lvguang2003@163.com (G.L.); 3 Department of Rehabilitation and Sports Medicine, Tianjin Medical University, 22 Qixiangtai Road, Heping District, Tianjin 300070, China; changhong@tmu.edu.cn; 4 Community Health Service Center for Shuangjie Town of Beichen District in Tianjin, Zhangwan Road, Shuangjie Town, Beichen District, Tianjin 300400, China; liuweitmu@126.com

**Keywords:** manganese, prediabetes, diabetes, older adult

## Abstract

Older adults can experience glucose metabolism dysfunction, and although manganese may help regulate glucose metabolism, there is little information regarding this association among older people. This cross-sectional study included 2402 Chinese adults who were ≥60 years old in 2013 (Tianjin, China), and evaluated the associations of serum manganese with prediabetes and diabetes. Serum manganese levels were measured using inductively coupled plasma mass spectrometry. Multivariable logistic regression models were used to evaluate the sex-specific associations of manganese levels with diabetes and prediabetes after adjusting for confounding factors (age, sex, life style factors, and health status). Based on the WHO criteria, prediabetes was observed in 15.1% of men and 13.4% of women, while diabetes was observed in 30.0% of men and 34.4% of women. In the final model, the odds ratios (95% confidence interval) for prediabetes according to manganese quartile were 1.000, 0.463 (0.269–0.798), 0.639 (0.383–1.065), and 0.614 (0.365–1.031) among men and 1.000, 0.773 (0.498–1.200), 0.602 (0.382–0.947), and 0.603 (0.381–0.953) among women (*p* for trend = 0.134 and 0.015, respectively). The lowest prevalence of diabetes among men occurred at a moderate range of serum manganese (*p* < 0.05). Therefore, appropriate serum manganese levels may help prevent and control prediabetes and diabetes.

## 1. Introduction

Diabetes mellitus is a major public health problem [1] and a leading cause of morbidity and mortality, with an estimated 346 million adults being affected worldwide in 2011 [2]. In China, the prevalence of diabetes is estimated to be 11.6% among adults [3], and the number and proportion of older adults are rapidly increasing in China and throughout the world [4]. The prevalence of diabetes also increases with age, and reaches 22.5% among adults who are ≥60 years old [3].

Trace elements, such as copper, zinc, iron, selenium, and manganese (Mn), are essential for human health and are involved in various metabolic and biological functions [5]. Deficiencies or excesses in these trace elements are usually related to human diseases. Mn is an essential trace element that is involved in normal immune functions, regulation of blood sugar and cellular energy, and protection against free radicals [6,7]. Mn is also a cofactor for several enzymatic systems and is required for normal insulin synthesis and secretion [8,9]. Moreover, some trace elements (e.g., Mn) can potentiate insulin’s action in reducing blood glucose levels [10]. However, it remains unclear whether Mn has effects in patients with diabetes.

Several studies have reported direct associations of Mn levels with diabetes [11,12,13,14,15,16,17], although it remains unclear whether Mn plays a positive or negative role. Furthermore, no studies have assessed the relationship between Mn levels and diabetes among seniors. The older age of this population also increases the risk of nutrient deficiencies, which can quickly lead to undernutrition and possibly contribute to the development of diseases [18]. Therefore, it is important to study the relationship between diabetes and Mn levels among older adults.

Prediabetes often precedes diabetes and is characterized by impaired fasting glucose (IFG) and/or impaired glucose tolerance (IGT) [19]. Prediabetes is also especially prevalent among older adults [4,20]. Compared to individuals with normal glucose metabolism, patients with prediabetes have a 5–15-fold higher risk of developing type 2 diabetes [21]. However, prediabetes is a reversible state [22], and the identification of risk factors for this state is an important step for the early prevention of diabetes. To the best of our knowledge, no studies have investigated the relationship between serum Mn levels and prediabetes among community-dwelling older adults. Therefore, we performed a cross-sectional study to determine whether serum Mn levels were associated with prediabetes and diabetes among community-dwelling ≥60-year-old adults.

## 2. Materials and Methods 

### 2.1. Study Population

This study included 3066 individuals who were ≥60 years old, living in the Shuangjie community of the Beichen district, and underwent health check-ups in 2013 at the Community Health Service Center (Beichen district, Tianjin, China). We invited all of these individuals to participate in a geriatric assessment, which included medical status, physical function, and cognitive function, and 2623 of the individuals completed the assessment (response rate: 85.6%). We excluded individuals with incomplete data (*n* = 178) and individuals whose serum Mn levels had not been measured (*n* = 43). Therefore, the final study population included 2402 participants (mean age: 67.5 ± 5.4 years; 40.1% men). The study’s protocol was approved by the Ethics Committee of Tianjin Medical University (TMUhMEC20120110), and all participants provided their written informed consent.

### 2.2. Serum Mn Measurements

Venous blood samples were collected after an overnight fast, were allowed to clot at room temperature, and were then centrifuged at 1000 rpm for 10 min. The serum was then transferred to Eppendorf tubes and stored at −20 °C until the analysis. Serum Mn levels were measured at the Tianjin Center for Disease Control and Prevention (Tianjin, China), based on a standardized protocol that incorporated sample processing, storage, and transportation to the analysis center. Serum Mn levels were analyzed using inductively coupled plasma mass spectrometry (Agilent7500cx; AGILENT TECHNOLOGIES INC., Santa Clara, CA, USA). The lower limit of detection for this assay was 0.035 μg/L, and scandium was used as the standard reference material for internal quality assurance and control (AGILENT TECHNOLOGIES INC). The inter-assay coefficients of variation ranged from 0.95% to 3.4% for serum Mn samples. 

### 2.3. Biochemical Assessments

Levels of fasting plasma glucose (FPG), total cholesterol (TC), triglycerides (TG), creatinine (CRE), and blood urea nitrogen (BUN) were determined using the Roche Modular P system (Roche Diagnostic Company, Switzerland). The creatinine clearance rate was calculated as ((140 − age in years) × weight in kg)/(72 × CRE in mg/mL), and 15% was subtracted from the calculated value for women.

### 2.4. Assessment of Diabetes and Prediabetes

Diabetes was defined by a self-reported physician’s diagnosis, use of hypoglycemic medication, or insulin treatment at the time of the interview. Among undiagnosed participants, diabetes was defined as FPG levels of ≥126 mg/dL (≥7.0 mmol/L) and prediabetes was defined as FPG levels of 110–126 mg/dL (6.1–7.0 mmol/L), based on the 1999 World Health Organization diagnostic criteria [23]. In this study, dysglycemia was defined as cases of both diabetes and prediabetes [24].

### 2.5. Assessment of Other Variables

The participants’ anthropometric characteristics (height and body weight) were recorded using a standardized protocol. Body mass index (BMI) was calculated as weight/height^2^ (kg/m^2^). A history of diabetes, hypertension, stroke, and/or coronary heart disease (CHD) was identified based on the participants’ responses regarding a physician’s diagnosis, taking any corresponding medication(s), or other treatment (current or previous). Information regarding smoking status (never, former smoker, and current smoker) and drinking status (never, daily drinker, and occasional drinker) were obtained using a single choice question questionnaire. Exercise status was assessed using a single self-reported question regarding frequency and duration of exercise during the last year: (1) “high” was defined as ≥3 sessions of ≥30 min each week; (2) “low” was defined as some exercise during the past year that did not qualify as “high”; and (3) “none” was defined as no exercise.

### 2.6. Statistical Analysis

Tests were performed to analyze the interactions between the serum Mn levels and sex in the final models. No significance interactions were found (*p* for interaction > 0.05). However, some studies have suggested that there are sex-specific differences in the homeostatic mechanisms that regulate blood Mn levels [25,26]. Furthermore, there are sex difference in the development, prevention, and management of diabetes [27,28,29]. Therefore, we categorized the participants according to sex for the reported of the results. The participants’ clinical and biochemical data were presented as mean ± standard deviation, number (%), or median and interquartile range for variables with a skewed distribution. Differences among the dysglycemia groups were evaluated using analysis of variance for continuous variables or using the *χ*^2^ test for categorical variables. Logistic regression analysis was used to determine the odds ratios (ORs) and 95% confidence intervals (CI) for the associations of serum Mn levels with prediabetes or diabetes. The ORs were calculated using a crude model and two adjusted models (Model A: adjusted for age, BMI, smoking status, drinking status, and exercise status; Model B: further adjusted for blood pressure, TG levels, TC levels, duration of diabetes, antidiabetic medication use, and a history of hypertension, cardiovascular disease, and/or stroke). Interactions between the serum Mn levels and sex were tested by the addition of the cross-product terms in the regression model. *p*-values for linear trends were calculated using the Mn level quartiles, and results were considered statistically significant at a two-tailed *p*-value of <0.05. All statistical analyses were performed using SPSS software (version 13.0; SPSS Inc., Chicago, IL, USA).

## 3. Results

Based on the World Health Organization criteria, we identified prediabetes among 14.1% of the participants (339/2402; men: 15.1% (146/964), women: 13.4% (193/1438)), and diabetes among 32.6% of the participants (783/2402; men: 30.0% (289/964), women: 34.4% (494/1438)). Serum Mn levels ranged from 0 µg/L to 46.34 μg/L, with an arithmetic mean of 9.49 μg/L and a geometric mean of 5.03 μg/L. The serum Mn concentrations were 2.91 μg/L, 5.79 μg/L, and 11.13 μg/L for the 25th, 50th, and 75th percentiles, respectively (Table 1).

### 3.1. Participant Characteristics

The participants’ characteristics according to sex are shown in Table 1. There were no sex-specific differences in age, BMI, FPG levels, serum Mn levels, or the prevalences of obesity and hypertension. Men had significantly higher values for blood pressure, BUN, and CRE, and significantly lower levels of TC and TG (*p* < 0.05). Men also exhibited higher prevalences of stroke, current smoker status, daily drinker status, and higher exercise status, while women exhibited a higher prevalence of diabetes and CHD.

The participants’ characteristics according to dysglycemia category are presented in Table 2. Participants with diabetes or prediabetes were more likely than normoglycemic participants to have higher values for blood pressure and BMI. In addition, men with prediabetes or diabetes had a higher prevalence of hypertension, while women exhibited higher values for systolic blood pressure, BMI, TG levels, hypertension, and obesity. Furthermore, older men with diabetes and older women with prediabetes were more likely to have exercised more during the last year, compared to normoglycemic participants (men: *p* < 0.001; women: *p* = 0.045). Men with diabetes were also more likely to have exercised more (vs. men with prediabetes), although the opposite trend was observed for women. Normoglycemic participants were significantly less likely to do any exercise, compared to participants with prediabetes or diabetes (*p* < 0.001). Women with prediabetes had lower serum Mn levels, compared to women with normoglycemia or diabetes (*p* = 0.001).

### 3.2. The Associations of Serum Mn Levels with Prediabetes and Diabetes

After adjusting for age, sex, BMI, lifestyle factors, and health status, participants with serum Mn levels in the higher quartiles exhibited a significant trend towards having lower ORs for the presence of prediabetes (*p* = 0.005, Table 3). When the higher quartiles (Q2 and Q3) were compared to the lowest quartile, the ORs for dysglycemia were 0.723 (95% CI: 0.569–0.918) and 0.704 (95% CI: 0.554–0.895), respectively (Table 3). No relationships were found between serum Mn levels and diabetes.

Table 4 shows the crude and adjusted associations of serum Mn levels (quartiles) with prediabetes and diabetes according to sex. In the crude analysis, women exhibited a lower prevalence of prediabetes with increasing serum Mn levels (*p* for trend = 0.009). After adjusting for age, BMI, smoking status, drinking status, and exercise status (Model A), the ORs for prediabetes according to serum Mn quartiles among women were 1.000, 0.622 (95% CI: 0.353–1.097), 0.471 (95% CI: 0.263–0.842), and 0.426 (95% CI: 0.232–0.784) (*p* for trend = 0.02). These results were unchanged when we adjusted for the other confounding factors in Model B. When the third Mn quartile was compared to the lowest quartile, the ORs in Model B for diabetes and dysglycemia were 0.675 (95% CI: 0.474–0.962) and 0.668 (95% CI: 0.489–0.913), respectively. However, there was no linear association between serum Mn levels and diabetes or dysglycemia among women (*p* for trend > 0.05). 

Among men, the crude ORs for prediabetes according to serum Mn quartile were 1.000, 0.462 (95% CI: 0.272–0.784, *p* = 0.004), 0.657 (95% CI: 0.400–1.080, *p* = 0.098), and 0.612 (95% CI: 0.370–1.011, *p* = 0.055); the OR was significantly lower for the second Mn quartile. The adjusted ORs for prediabetes in the second Mn quartile were 0.455 (95% CI: 0.267–0.776, *p* = 0.004) for Model A and 0.463 (95% CI: 0.269–0.798, *p* = 0.003) for Model B (vs. the first quartile). Similar results were observed in the diabetes and dysglycemia (prediabetes or diabetes) analyses, with the crude OR for the second Mn quartile being the lowest (OR for diabetes: 0.618, 95% CI: 0.406–0.939, *p* = 0.024; OR for dysglycemia: 0.551, 95% CI: 0.383–0.793, *p* = 0.001). These lower ORs for diabetes and dysglycemia remained significant in both Model A and Model B (Table 4). There was no linear association between serum Mn levels and prediabetes or diabetes among men (*p* for trend > 0.05).

We also assessed the relationship between log-transformed Mn levels and the prevalences of prediabetes and diabetes. In the crude model, increasing log-transformed Mn levels exhibited a significant inverse relationship with the prevalence of prediabetes (*p* = 0.003 for the total population; *p* = 0.040 for men; and *p* = 0.033 for women). Even after the adjustment in the final model (Model B), the significant inverse relation remained unchanged (total population: OR: 0.705, 95% CI: 0.555–0.896, *p* = 0.004; men: OR: 0.673, 95% CI: 0.465–0.975, *p* = 0.036; women: OR: 0.732, 95% CI: 0.531–0.974, *p* = 0.046). However, no inverse relationship between log-transformed Mn levels and diabetes or dysglycemia was observed in the models.

## 4. Discussion

This study examined the associations of serum Mn levels with prediabetes and diabetes among community-dwelling adults who were ≥60 years old. Although several studies have investigated the relationships between levels of trace elements and diabetes, few studies have evaluated the relationship between serum Mn levels and diabetes [11,12]. Moreover, no studies have evaluated the relationship between serum Mn levels and prediabetes according to sex. Thus, the strength of this large community-based study is that we were able to adjust for several confounding factors, and our results suggest that high serum Mn levels are independently associated with a lower prevalence of prediabetes among older women. Moderate serum Mn levels (the second quartile) were independently associated with lower prevalences of prediabetes and diabetes among older men.

Four small-scale cross-sectional studies have evaluated the relationships between levels of trace elements (including plasma Mn) and diabetes among American [30], Austrian [31], Turkish [32], and Czech [12] individuals. However, these studies did not find a statistically significant relationship between Mn levels and diabetes. In contrast, several other epidemiological studies have found an inverse relationship between Mn levels and diabetes among Russian [13], Pakistani [14], Italian [15], Nigerian [33], and Korean [11] individuals. Additionally, several other cross-sectional studies have found a positive association between Mn levels and diabetes among Russian [16], Mexican [17], and Turkish [34] individuals. Only two of these studies evaluated the general population [11,12], and only one study targeted older people [12]. Thus, our study provides the first data regarding the associations of Mn levels with prediabetes and diabetes among older Chinese individuals. Our results indicate that there is an inverse linear relationship between Mn levels and prediabetes among older women (but no linear relationship between Mn levels and diabetes), and that an association exists between moderate Mn levels and prediabetes/diabetes among older men. Although the reason for this discrepancy remains unclear, it is likely that differences in regions, dietary habits, race, and age may contribute. For example, age is associated with declining Mn levels [25]. Moreover, food is the main source of Mn in the general population [35] and different foods contain different levels of Mn, dietary manganese intake levels range from 1.38 mg/day to 6.8 mg/day, based on country-specific differences dietary patterns [36,37,38,39,40]. 

In the present study, women exhibited a negative linear association of serum Mn levels with prediabetes, although this association was not observed among men. This difference is likely related to circulating Mn levels being regulated by complex homeostatic mechanisms, and these levels may vary according to sex, age, and other variables [25]. Some studies have revealed that estrogen use did not affect Mn status among adult women [41], and hormonal replacement therapy does not influence serum Mn status among postmenopausal women [42]. Another study of dietary Mn intake revealed that women absorb significantly more Mn (vs. men) from a diet with adequate Mn [26]. That study also found that the biological half-life of Mn was significantly shorter in women, compared to men. These findings suggest that men and women exhibit differences in Mn absorption and metabolism, which may partially explain the sex differences that we observed in the present study. In addition, a Spanish study showed that inadequate Mn intake might favor insulin resistance among girls, which suggests that ensuring adequate Mn intake may help prevent insulin resistance and type 2 diabetes among girls [43]. Therefore, these results indicate that altered Mn metabolism is more important for women, who might be more sensitive to Mn deficiency, and we speculate that the sex differences may be related to differences in Mn metabolism, rather than hormonal differences. Nevertheless, further research is needed to explain the sex-specific differences in the association of Mn levels with prediabetes and diabetes. 

We did not detect any sex-specific differences in serum Mn levels for participants with normoglycemia, prediabetes, or diabetes, although several other studies have reported higher blood Mn levels among women (vs. men) [44,45,46]. However, the Canadian and South Korean studies did not clarify the differences between men and women who were ≥60 years old [44,46]. In contrast, the Swedish study reported a small difference between men and women who were ≥70 years old (7.3 mg/L vs. 7.9 mg/L, respectively) [45]. Nevertheless, our results are consistent with those from a Czech study [12]. Therefore, we speculate that sex-specific differences in serum Mn levels may also be related to age, which is supported by an American study that found sex-specific differences in blood Mn levels decreased with age [25]. In addition, a Korean study found that blood Mn levels decreased in postmenopausal women compared to that in premenopausal women after adjusting for various factors [47], which suggests that hormone may partly explain the insignificant sex-specific differences in blood Mn levels between men and postmenopausal women in our study. 

We only observed a linear association between serum Mn levels and prediabetes among women, although all of the participants were ≥60 years old and may have had diabetes for an extended period of time. Chronic diabetes is typically managed using medication and diet control, which might affect Mn metabolism. Thus, diabetes-associated changes in diet and medication may confound analyses of Mn levels and diabetes, which could also explain the different conclusions from the various studies. In contrast, individuals with prediabetes are not yet receiving hypoglycemic medication or dietary adjustment, which may provide a more accurate state for evaluating changes in Mn metabolism. 

Appropriate levels of Mn are required for insulin synthesis and secretion, while Mn deficiency can lead to poor glucose metabolism [48]. High serum Mn levels might also be involved in oxidative stress protection and increase insulin secretion. Some studies have found that blood Mn levels range from 1.6 μg/L to 62.5 μg/L, with changes according to sex, age, race/ethnicity and other blood mineral levels [24,49]. An animal study also revealed that manganese superoxide dismutase metalation and activity in normal mice can be augmented using Mn supplementation, and that Mn treatment increased insulin secretion to improve glucose tolerance during periods of dietary stress [50]. Nevertheless, Mn is a trace element with unclear essential and adverse effects, as excess Mn is related to neurotoxic effects [51], although the negative effects are only induced after ingestion of contaminated food or water [15]. There is only adequate intake in China (4.5 mg/day), as the tolerable upper intake limit is 11 mg/day [52], which may be related to insufficient information regarding Mn requirements. Thus, optimal Mn intake levels are not known, and our results might suggest the sex differences should be considered when recommending dietary intake in future guidelines. 

This study had several limitations. First, the cross-sectional design precludes any conclusions regarding the causality or direction of the associations between serum Mn levels and prediabetes or diabetes. Second, although we adjusted for various confounding factors, we cannot exclude the possibility that the associations of serum Mn levels with prediabetes or diabetes are affected by other dietary factors, medications (such as HRT), and/or Mn supplementation. In addition, we cannot adjust for HbA1c levels, as these data were not obtained during the health screening. Third, we did not consider other trace elements (e.g., iron or zinc) that can affect Mn levels, and some studies have found that individuals with low iron status have higher blood Mn levels [43,53]. This relationship may also explain the sex-specific differences in Mn metabolism. Therefore, a well-designed prospective study is needed to validate our findings, and we hope to provide additional data in our future studies. 

## 5. Conclusions 

This study revealed that serum Mn levels are likely associated with prediabetes and diabetes among older Chinese people. For example, higher serum Mn levels were independently associated with a lower prevalence of prediabetes among older community-dwelling women, which suggests that a Mn-rich diet may be useful in this population. Furthermore, we found that moderate serum Mn levels were associated with lower prevalence of prediabetes and diabetes among older men, which suggests that a normal dietary intake of Mn may be beneficial in this population. Nevertheless, there may be sex-specific roles for Mn in prediabetes and diabetes, and prospective studies of older adults are needed to confirm these findings.

## Figures and Tables

**Table 1:** Descriptive participants’ characteristics by sex.

Variables	Total	Men	Women	*p*-Value
*N*	2402	964	1438	
Age, year	67.54 ± 5.40	67.49 ± 5.41	67.58 ± 5.38	0.684
SBP, mmHg	136.68 ± 16.57	137.54 ± 16.99	136.10 ± 16.26	0.037
DBP, mmHg	82.06 ± 8.82	83.01 ± 9.64	81.42 ± 8.17	<0.001
BMI, kg/m^2^	26.42 ± 3.54	26.29 ± 3.28	26.51 ± 3.71	0.134
Blood index				
TC, mmol/L	5.37 ± 1.03	5.05 ± 0.93	5.59 ± 1.04	<0.001
TG, mmol/L	1.51 (1.10, 2.10)	1.40 (1.00, 1.96)	1.60 (1.20, 2.20)	<0.001
FPG, mmol/L	6.5 ± 11.90	6.49 ± 1.77	6.53 ± 1.98	0.587
BUN, mmol/L	5.81 ± 1.63	6.01 ± 1.61	5.68 ± 1.66	<0.001
CCr, min/L	60.14 ± 22.69	64.51 ± 16.03	57.21 ± 25.82	<0.001
CRE, μmol/L	97.64 ± 34.04	108.23 ± 44.80	90.54 ± 21.58	<0.002
Smoking status, % (*n*)				
Current smoker	19.2 (462)	32.0 (308)	10.7 (154)	<0.001
Former smoker	3.5 (84)	6.0 (58)	1.8 (26)	<0.001
Nonsmoker	77.3 (1856)	62.0 (598)	87.5 (1258)	<0.001
Drinking status, % (*n*)				
Daily drinker	7.1 (170)	16.7 (161)	0.6 (9)	<0.001
Occasional drinker	4.8 (116)	11.4 (110)	0.4 (6)	<0.001
Nondrinker	88.1 (2116)	71.9 (693)	99.0 (1423)	<0.001
Exercise status, % (*n*)				
High	51.2 (1229)	55.7 (537)	48.1 (692)	0.001
Low	16.5 (396)	14.4 (139)	17.9 (257)	0.029
None	32.3 (777)	29.9 (288)	34.0 (489)	0.038
Serum Mn, μg/L	5.79 (2.91, 11.13)	6.02 (2.86, 11.17)	5.64 (2.95, 11.10)	0.694
History of Disease				
Obesity, % (*n*)	29.8 (715)	28.4 (274)	30.7 (441)	0.238
Diabetes, % (*n*)	28.4 (682)	25.6 (247)	30.3 (435)	0.014
Hypertension, % (*n*)	72.5 (1742)	72.6 (700)	72.5 (1042)	0.972
Stroke, % (*n*)	4.6 (111)	7.1 (68)	3.0 (43)	<0.001
CHD, % (*n*)	14.7 (353)	10.9 (105)	17.2 (248)	<0.001

Abbreviations: SBP, systolic blood pressure; DBP, diastolic blood pressure; BMI, body mass index; TC, total cholesterol; TG, triglyceride; FPG, fasting plasma glucose; BUN, blood urea nitrogen; CCr, creatinine clearance; CRE, creatinine; CHD, coronary heart disease; Mn, manganese; *p* values were calculated from Chi-square test for categorical variables and Student’s *t* tests or the Wilcoxon rank-sum test were used for between-group comparisons of normally distributed and skewed data, respectively; All continuous variables were presented as means ± SD or medians (interquartile ranges), and all categorical variables were presented as number (proportions).

**Table 2:** Participants’ characteristics according to sex and categories of dysglycemia.

	Men				Women			
Variables	Normoglycemia (*n* = 529)	Prediabetes (*n* = 146)	Diabetes (*n* = 289)	*p*-Value	Normoglycemia (*n* = 751)	Prediabetes (*n* = 193)	Diabetes (*n* = 494)	*p*-Value
Age, year	67.41 ± 5.41	66.97 ± 5.24	67.90 ± 5.51	0.208	67.29 ± 5.05	67.27 ± 5.23	68.14 ± 5.87	0.106
SBP, mmHg	136.00 ± 17.24	141.85 ± 8.64 *	138.19 ± 15.23 ^#^	0.001	134.44 ± 15.93	137.54 ± 17.33 *	138.07 ± 16.08 *	<0.001
DBP, mmHg	82.99 ± 9.71	84.97 ± 10.00 *	82.03 ± 9.18 ^#^	0.011	81.10 ± 8.05	82.17 ± 8.70	81.49 ± 8.14	0.325
BMI, kg/m^2^	26.04 ± 3.36	26.62 ± 3.33	26.59 ± 3.07 *	0.03	26.09 ± 3.64	26.96 ± 4.04 *	26.96 ± 3.61 *	<0.001
Blood index								
TC, mmol/L	5.01 ± 0.91	5.15 ± 0.92	5.08 ± 0.95	0.214	5.59 ± 1.01	5.71 ± 1.03	5.54 ± 1.09	0.148
TG, mmol/L	1.37 (1.00, 1.90)	1.48 (1.07, 2.10)	1.45 (1.05, 2.00)	0.087	1.59 (1.20, 2.10)	1.71 (1.30, 2.41) *	1.66 (1.20, 2.30)	0.024
FPG, mmol/L	5.47 ± 0.43	6.43 ± 0.26 *	8.38 ± 2.14 *^,#^	<0.001	5.43 ± 0.44	6.43 ± 0.26 *	8.24 ± 2.51 *^,#^	<0.001
BUN, mmol/L	5.97 ± 1.53	5.97 ± 1.60	6.12 ± 1.74	0.414	5.61 ± 1.49	5.64 ± 1.71	5.78 ± 1.80	0.190
CRE, μmol/L	107.96 ± 46.23	107.01 ± 16.29	109.35 ± 51.53	0.858	89.48 ± 14.63	90.39 ± 15.90	92.22 ± 30.48	0.090
Smoking status, %(*n*)								
Current smoker	33.5 (177)	33.6 (49)	28.4 (82)	0.297	11.5 (86)	9.8 (19)	9.9 (49)	0.636
Former smoker	5.3 (28)	8.2 (12)	6.2 (18)	0.414	1.5 (11)	1.0 (2)	2.6 (13)	0.219
Nonsmoker	61.2 (324)	58.2 (85)	65.4 (189)	0.297	87.1 (654)	89.1 (172)	87.4 (432)	0.748
Drinking status, %(*n*)								
Daily drinker	18.3 (97)	16.4 (24)	13.8 (40)	0.256	0.9 (7)	0.5 (1)	0.2 (1)	0.274
Occasional drinker	10.4 (55)	15.8 (23)	11.1 (32)	0.193	0.5 (4)	1.0 (2)	0 (0)	0.129
Nondrinker	71.3 (377)	67.8 (99)	75.1 (217)	0.251	98.5 (740)	98.4 (190)	99.8 (493)	0.076
Exercise status, %(*n*)								
High	50.1 (265)	55.5 (81)	66.1 (191) *^,#^	<0.001	45.9 (345)	56.0 (108) *	48.4 (239) ^#^	0.045
Low	14.9 (79)	14.4 (21)	13.5 (39)	0.855	16.6 (125)	21.8 (42)	18.2 (90)	0.247
None	35.0 (185)	30.1 (44)	20.4 (59) *^,#^	<0.001	37.4 (281)	22.3 (43) *	33.4 (165) ^#^	<0.001
Serum Mn, μg/L	5.92 (3.05, 10.25)	5.66 (2.15, 11.98)	6.24 (2.56, 12.74)	0.577	6.16 (3.28, 11.66)	4.49 (2.39, 8.69) *	5.56 (3.02, 11.14) ^#^	0.001
Low, %(*n*)	49.9 (264)	52.1 (76)	44.3 (128)	0.201	49.0 (368)	58.0 (112)	50.2 (248)	0.080
Normal, %(*n*)	48.2 (255)	47.3 (69)	53.3 (154)	0.316	50.1 (376)	41.5 (80)	49.0 (242)	0.099
High, %(*n*)	1.9 (10)	0.7 (1)	2.4 (7)	0.449	0.9 (7)	0.5 (1)	0.8 (4)	0.851
Disease								
Obesity, %(*n*)	25.5 (135)	32.2 (47)	31.8 (92)	0.088	26.1 (196)	38.3 (74) *	34.6 (171) *	<0.001
Hypertension, %(*n*)	67.5 (357)	75.3 (110)	80.6 (233) *	<0.001	64.7 (486)	74.1 (143) *	83.6 (413) *^,#^	<0.001
Stroke, %(*n*)	6.2 (33)	5.5 (8)	9.3 (27)	0.183	2.3 (17)	1.0 (2)	4.9 (24) *^,#^	0.007
CHD, %(*n*)	11.5 (61)	7.5 (11)	11.4 (33)	0.368	16.8 (126)	13.5 (26)	19.4 (96)	0.157

Prediabetes is defined by having fasting plasma glucose (FPG) levels ≥110 mg/dL (6.1 mmol/L) but <126 mg/dL (7.0 mmol/L); diabetes is defined by self-reported or FPG ≥126 mg/dL (7.0 mmol/L); Abbreviations: SBP, systolic blood pressure; DBP, diastolic blood pressure; BMI, body mass index; TC, total cholesterol; TG, triglyceride; FPG, fasting plasma glucose; BUN, blood urea nitrogen; CRE, creatinine; CHD, coronary heart disease; Obtained by using ANOVA for continuous variables and chi-square for variables of proportion; All continuous variables were presented as means ± SD or medians (interquartile ranges), and all categorical variables were presented as number (proportions); * normoglycemia compare with prediabetes & diabetes; ^#^ prediabetes compare with diabetes (*p* < 0.05).

**Table 3:** Adjusted odds ratios (95% confidence interval) of the association between serum Mn quartiles and dysglycemia (prediabetes and diabetes).

Variables	Serum Mn Quartiles				*p* for Trend
	Q1	Q2	Q3	Q4	
Prediabetes					
Mn, μg/L (serum level)	0~2.858	2.858~5.332	5.332~9.676	9.676~46.000	
Number at risk	404	405	405	405	
Number with prediabetes	110	77	78	74	
Crude	1.000	0.610 (0.438, 0.849)	0.630 (0.453, 0.876)	0590 (0.423, 0.824)	0.003
Model A	1.000	0.607 (0.434, 0.849)	0.633 (0.454, 0.883)	0.607 (0.433, 0.851)	0.005
Model B	1.000	0.625 (0.446, 0.877)	0.619 (0.442, 0.867)	0.614 (0.437, 0.863)	0.005
Diabetes					
Mn, μg/L (serum level)	0~2.952	2.952~5.534	5.534~10.384	10.384~46.340	
Number at risk	515	517	515	516	
Number with diabetes	205	179	180	219	
Crude	1.000	0.801 (0.622, 1.031)	0.813 (0.631, 1.046)	1.115 (0.870, 1.429)	0.387
Model A	1.000	0.800 (0.619, 1.035)	0.806 (0.623, 1.043)	1.141 (0.887, 1.469)	0.316
Model B	1.000	0.824 (0.627, 1.082)	0.762 (0.579, 1.003)	1.150 (0.881, 1.501)	0.413
Prediabetes + Diabetes					
Mn, μg/L (serum level)	0~2.836	2.836~5.429	5.429~10.092	10.092~46.340	
Number at risk	601	601	600	600	
Number with dysglycemia	309	257	263	293	
Crude	1.000	0.706 (0.562, 0.886)	0.737 (0.588, 0.926)	0.902 (0.719, 1.131)	0.463
Model A	1.000	0.708 (0.562, 0.892)	0.738 (0.586, 0.929)	0.916 (0.728, 1.152)	0.547
Model B	1.000	0.723 (0.569, 0.918)	0.704 (0.554, 0.895)	0.909 (0.717, 1.153)	0.413

Mn: manganese; ORs were determined from logistic regression analyses for the quartiles of serum manganese, comparing participants with dysglycemia (prediabetes and/or diabetes) to those with normoglycemia; Crude: no adjustment; Model A: adjusted for age, sex, body mass index (BMI), smoking status, drinking status, exercise status; Model B: additionally adjusted for blood pressure (BP), triglyceride (TG), total cholesterol (TC), duration of diabetes, antidiabetic medication use and history of hypertension, stroke, and coronary heart disease (CHD); Adjusted odds ratio; 95% CI in parentheses.

**Table 4:** Adjusted odds ratios (95% confidence interval) of the association between serum Mn quartiles and dysglycemia (prediabetes and/or diabetes) according to sex.

Variables	Men					Women				
	Q1	Q2	Q3	Q4	*p* for Trend	Q1	Q2	Q3	Q4	*p* for Trend
Prediabetes										
Serum Mn (μg/L)	0~2.52	2.52~5.12	5.13~9.85	9.86~46.00		0~2.91	2.91~5.65	5.65~11.06	11.06~15.49	
Number at risk	168	169	169	169		236	236	236	236	
Number with prediabetes	49	27	36	34		62	49	42	40	
Crude	1.000	0.462 (0.272, 0.784)	0.657 (0.400, 1.080)	0.612 (0.370, 1.011)	0.126	1.000	0.578 (0.332, 1.006)	0.473 (0.266, 0.839)	0.383 (0.211, 0.699)	0.009
Model A	1.000	0.455 (0.267, 0.776)	0.657 (0.398, 1.083)	0.615 (0.370, 1.021)	0.137	1.000	0.622 (0.353, 1.097)	0.471 (0.263, 0.842)	0.426 (0.232, 0.784)	0.020
Model B	1.000	0.463 (0.269, 0.798)	0.639 (0.383, 1.065)	0.614 (0.365, 1.031)	0.134	1.000	0.773 (0.498, 1.200)	0.602 (0.382, 0.947)	0.603 (0.381, 0.953)	0.015
Diabetes										
Serum Mn (μg/L)	0~2.92	2.92~5.74	5.74~10.30	10.30~46.34		0~2.95	2.95~5.45	5.45~10.38	10.38~26.38	
Number at risk	204	205	204	205		311	312	311	311	
Number with diabetes	76	55	69	89		130	124	109	131	
Crude	1.000	0.618 (0.406, 0.939)	0.867 (0.578, 1.302)	1.267 (0.852, 1.883)	0.104	1.000	0.918 (0.667, 1.264)	0.751 (0.543, 1.039)	1.013 (0.737, 1.393)	0.868
Model A	1.000	0.642 (0.418, 0.987)	0.860 (0.567, 1.303)	1.283 (0.854, 1.925)	0.113	1.000	0.905 (0.654, 1.254)	0.734 (0.527, 1.020)	1.020 (0.738, 1.410)	0.808
Model B	1.000	0.659 (0.414, 0.992)	0.850 (0.544, 1.326)	1.361 (0.884, 2.096)	0.086	1.000	0.989 (0.701, 1.396)	0.675 (0.474, 0.962)	1.007 (0.714, 1.420)	0.649
Prediabetes + Diabetes										
Serum Mn (μg/L)	0~2.80	2.83~5.58	5.61~10.20	10.22~46.34		0~2.86	2.86~5.33	5.34~9.99	9.99~26.38	
Number at risk	241	241	241	241		358	361	360	359	
Number with dysglycemia	122	87	105	121		186	171	159	171	
Crude	1.000	0.551 (0.383, 0.793)	0.753 (0.526, 1.078)	0.984 (0.688, 1.406)	0.664	1.000	0.832 (0.621, 1.115)	0.732 (0.545, 0.981)	0.841 (0.627, 1.128)	0.171
Model A	1.000	0.574 (0.397, 0.832)	0.760 (0.528, 1.095)	0.992 (0.690, 1.425)	0.671	1.000	0.823 (0.611, 1.108)	0.726 (0.539, 0.978)	0.852 (0.633, 1.147)	0.206
Model B	1.000	0.572 (0.388, 0.844)	0.744 (0.509, 1.088)	0.987 (0.677, 1.439)	0.728	1.000	0.888 (0.652, 1.208)	0.668 (0.489, 0.913)	0.847 (0.622, 1.153)	0.117

Mn: manganese; ORs were determined from logistic regression analyses for the quartiles of serum manganese, comparing participants with dysglycemia (prediabetes and/or diabetes) to those with normoglycemia; Crude: no adjustment; Model A: adjusted for age, body mass index (BMI), smoking status, drinking status, exercise status; Model B: additionally adjusted for blood pressure (BP), triglyceride (TG), total cholesterol (TC), duration of diabetes, antidiabetic medication use, and history of hypertension, stroke, and coronary heart disease (CHD); Adjusted odds ratio; 95% CI in parentheses.
